# Kidney‐Targeted Cilastatin Nanoparticles Inhibit DPEP1‐Mediated Ferroptosis for Acute Kidney Injury Therapy

**DOI:** 10.1002/advs.77090

**Published:** 2026-08-13

**Authors:** Wanbing Qin, Jiaqi Huang, Manting Zhang, Wanchun Yang, Mingwei Xu, Chi Jiang, Jiekai Li, Jinjuan Wen, Qibin Huang, Shiqi Wen, Weidong Wang, Junbing He, Qinghua Liu

**Affiliations:** ^1^ Jieyang Medical Research Center Jieyang People's Hospital Jieyang Guangdong China; ^2^ Department of Pathophysiology Zhongshan School of Medicine Sun Yat‐sen University Guangzhou China; ^3^ Department of Nephrology The First Affiliated Hospital Sun Yat‐sen University Guangzhou Guangdong China; ^4^ Department of Nephrology Jieyang People's Hospital Jieyang Guangdong China; ^5^ Guangdong Provincial Key Laboratory of Nephrology NHC Key Laboratory of Clinical Nephrology (Sun Yat‐sen University) Guangzhou China

**Keywords:** acute kidney injury, anti‐ferroptotic therapy, dipeptidase 1, polydopamine nanoparticle, targeted therapy

## Abstract

Cisplatin‐induced acute kidney injury (CI‐AKI) is a frequent, dose‐dependent complication in patients receiving cisplatin chemotherapy, and effective targeted treatments remain unavailable. The development of targeted therapies for CI‐AKI may improve the efficacy of chemotherapy while mitigating renal toxicity. Given that ferroptosis is a key pathological mechanism in CI‐AKI, targeted drug delivery systems against this process represent a promising therapeutic strategy. Dipeptidase 1 (DPEP1) is highly expressed in renal proximal tubular brush borders and has been associated with ferroptosis regulation and tubular injury. In this study, mesoporous polydopamine nanoparticles (MPDA NPs) were engineered for renal‐targeted delivery of cilastatin sodium (CTT), a specific DPEP1 inhibitor, through L‐serine (L‐Ser) surface modification (CTT/MPDA@L‐Ser NPs). In vitro experiments demonstrated that CTT/MPDA@L‐Ser NPs inhibited the expression of DPEP1 to upregulate the expression of the ferroptosis‐suppressing protein glutathione peroxidase 4 (GPX4), thereby suppressing ferroptosis pathways. In AKI mice, CTT/MPDA@L‐Ser NPs exhibited selective renal accumulation, significantly ameliorating serum creatinine and blood urea nitrogen levels. Collectively, CTT/MPDA@L‐Ser NPs exhibited potent therapeutic efficacy against AKI, presenting a promising strategy for targeted AKI management.

## Graphical abstract

In this study, MPDA NPs were engineered to encapsulate CTT. To further increase renal accumulation of CTT, L‐serine was conjugated to the surface of the MPDA nanoparticles (CTT/MPDA@L‐Ser NPs). CTT/MPDA@L‐Ser NPs inhibit DPEP1 activity, thereby restoring ferroptosis defense mechanisms through upregulation of GPX4 and SLC7A11 expression, ultimately mitigating cisplatin‐induced AKI pathology.

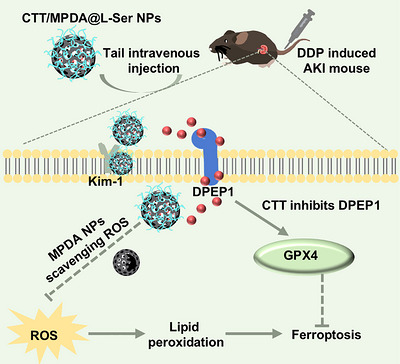

## 1 Introduction

As a first‐line drug for lung cancer, cisplatin is associated with severe adverse effects. Notably, cisplatin‐induced acute kidney injury (CI‐AKI) is a common dose‐dependent complication in treated patients. Acute kidney injury (AKI) refers to a rapid decline in renal function over a short period (hours to weeks), manifested by a rapid increase in serum creatinine levels, reduced urine output, and subsequent accumulation of creatinine and urea [1, 2, 3]. AKI has a high incidence and mortality rate among kidney diseases, with reported incidence rates of 10%–15% in hospitalized patients and up to 50% in intensive care units [4, 5, 6, 7]. However, current clinical management of CI‐AKI is limited to cisplatin dosage adjustment or intravenous hydration to enhance renal perfusion [8]. These nonspecific interventions may compromise chemotherapeutic outcomes. Consequently, developing targeted agents that enable concomitant prevention and prompt mitigation of CI‐AKI without impeding therapeutic efficacy represents an urgent unmet need.

The pathogenesis of CI‐AKI involves excessive generation of reactive oxygen species (ROS), inflammatory cytokine storms, apoptosis, and ferroptosis [9, 10, 11]. Ferroptosis is an oxidative, nonapoptotic form of cell death characterized by iron‐dependent accumulation of lipid peroxides [12]. It is typically associated with altered expression of ferroptosis‐related proteins, such as downregulation of glutathione peroxidase 4 (GPX4) and solute carrier family 7 member 11 (SLC7A11) protein, as well as changes in mitochondrial morphology, including mitochondrial shrinkage, increased membrane density, loss of cristae, and outer membrane rupture [13]. It has been implicated in various pathological processes, including cancer, neurodegenerative diseases, ischemic organ injury, and kidney diseases. Substantial evidence implicates ferroptosis in the pathogenesis of AKI [14, 15, 16], underscoring the therapeutic imperative for targeted pharmacological modulation of renal ferroptosis pathways.

Dipeptidase 1 (DPEP1) is a zinc‐dependent metalloprotease expressed at the renal brush border [17]. It primarily participates in the metabolism of glutathione and its conjugates (e.g., leukotrienes) [18, 19, 20], and some studies have shown that DPEP1 can regulate ferroptosis. Guan et al. reported that a single genetic locus regulates DPEP1/CHMP1A expression, thereby modulating the ferroptosis pathway and influencing the progression of kidney disease [21]. Extensive experimental evidence has demonstrated that the knockout of DPEP1 inhibits ferroptosis induced by folic acid, cisplatin, and other agents, significantly reducing ferroptosis‐related protein expression in the kidney. However, gene knockout therapy is difficult in the clinic, making the development of small‐molecule inhibitors targeting DPEP1 a promising strategy for anti‐ferroptosis therapy.

Cilastatin sodium (CTT) is a specific small‐molecule inhibitor of DPEP1. Studies have shown that CTT can inhibit DPEP1, thereby alleviating renal tubular damage [22]. CTT has been widely used in clinical practice as an antibacterial synergist, indicating that it has good safety. Recent advances in kidney‐targeted therapeutics have emphasized the importance of rational delivery system design for renal diseases. Deng et al. noted that kidney‐targeted nanoplatforms can deliver therapeutics directly to specific regions of the kidney, thereby improving drug efficacy and reducing off‐target effects. The same review summarized passive targeting based on renal pathophysiology and active targeting using ligand‐receptor recognition as two major strategies for kidney‐targeted nanoplatforms [23]. Patel et al. highlighted that effective kidney‐targeted therapeutics require careful consideration of both the therapeutic cargo and the delivery carrier. They further summarized recent progress in viral and non‐viral delivery vehicles for kidney‐targeted therapy, including strategies that leverage endogenous biological mechanisms, cross‐species and route‐of‐administration screening, and artificial intelligence to improve kidney selectivity. These findings support the importance of rational carrier‐cargo design in kidney‐targeted therapeutic development [24]. These perspectives provide the rationale for constructing a kidney‐targeted nanoplatform to improve the delivery efficiency of CTT in CI‐AKI. Therefore, the key challenge remaining is achieving renal accumulation of CTT. Thus, in this study, mesoporous polydopamine nanoparticles (MPDA NPs) were designed for CTT delivery. Polydopamine (PDA) is a biomimetic nanomaterial formed by self‐polymerization of dopamine and is widely used in biomedicine, energy, and catalysis because of its unique chemical properties, excellent biocompatibility, and versatility. PDA nanoparticles have many active bonds and are easily modified on the surface, which improves the versatility of PDA materials [25]. Additionally, it has been suggested that PDA can also scavenge ROS to play an antioxidant role [26].

**FIGURE 1:**
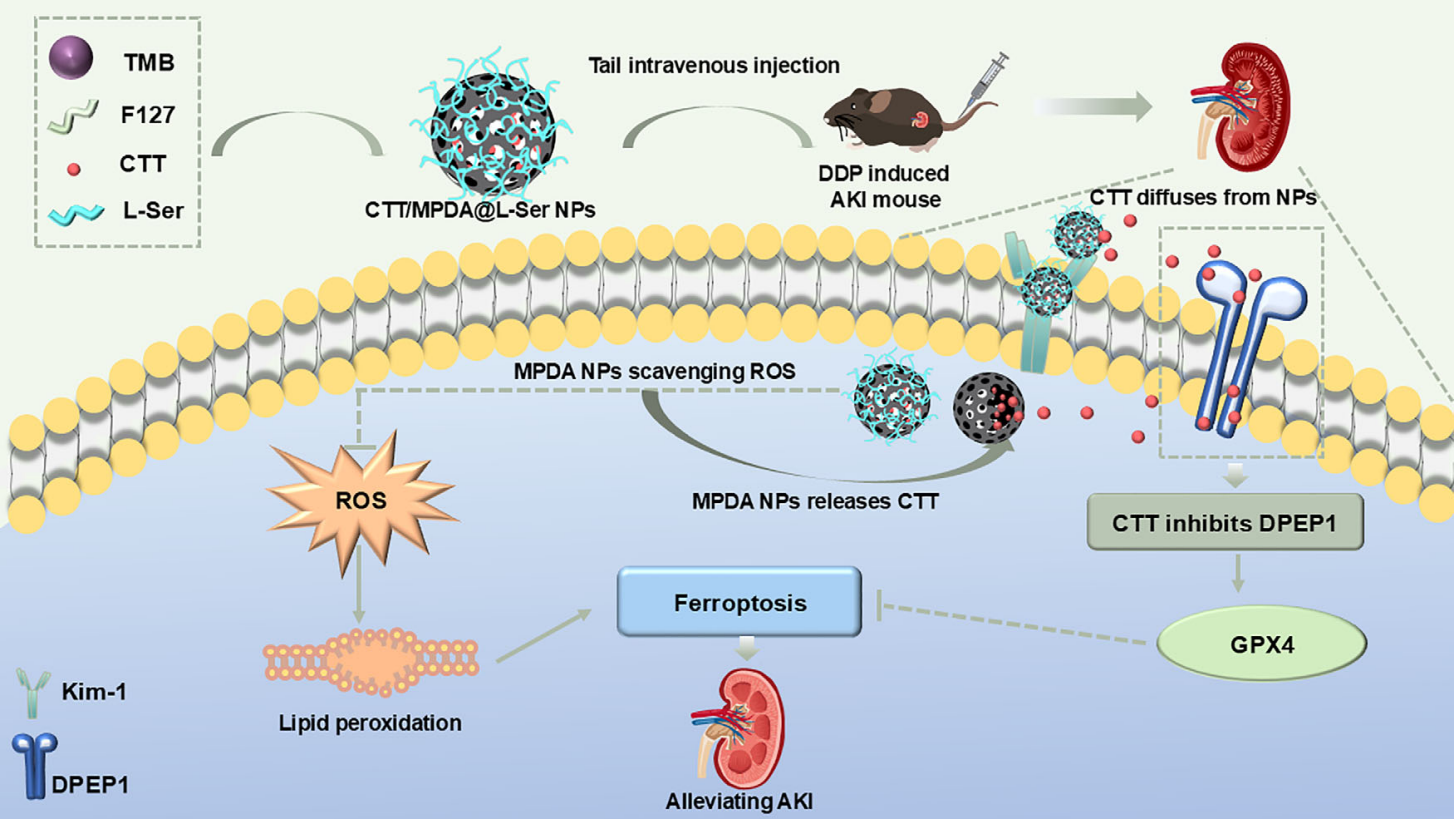
Schematic illustration of CTT/MPDA@L‐Ser NPs drug delivery system for CI‐AKI therapy.

In this study, MPDA NPs were engineered to encapsulate CTT. Research has indicated that L‐serine (L‐Ser) can target kidney injury molecule‐1 (KIM‐1) receptors on the surface of injured kidneys during AKI [1, 27]. To further increase renal accumulation of CTT, L‐serine was conjugated to the surface of the MPDA nanoparticles. As illustrated in Figure 1, systemically administered L‐serine‐modified nanoparticles selectively targeted KIM‐1 on renal tubules [1, 28, 29], enabling renal‐specific accumulation and localized CTT release. The liberated CTT inhibits DPEP1 activity, thereby restoring ferroptosis defense mechanisms through upregulation of GPX4 and SLC7A11 expression, ultimately mitigating cisplatin‐induced AKI pathology. Furthermore, MPDA scavenges ROS, thereby reducing lipid peroxidation and consequently suppressing ferroptosis.

## 2 Materials and Methods

### 2.1 Materials, Cells, and Animals

Dopamine hydrochloride (DA), 1,3, 5‐Trimethylbenzene (TMB), and Pluronic F‐127 (F127) were purchased from Macklin (Shanghai, China), Aladdin Reagent (Shanghai, China), and Sigma (Shanghai, China), respectively. Cisplatin and CTT were purchased from Aladdin Reagent (Shanghai, China). DPEP1 polyclonal antibody was purchased from Cusabio (Wuhan, China), GPX4 Polyclonal antibody and SLC7A11/xCT Polyclonal antibody were purchased from Proteintech (Wuhan, China). Kim‐1 antibody was obtained from ZEN‐Bioscience (Chengdu,China). DCFH‐DA was purchased from Biyuntian Biotechnology (Shanghai, China). Glutathione (GSH) assay kit and Malondialdehyde (MDA) test kit were purchased from Jiancheng Bioengineering Institute (Nanjing, China). Cell counting kit (CCK‐8) was obtained from Seven (Beijing, China).

HK‐2 cells and HK‐2 cell culture medium were obtained from Procell (Wuhan, China). The cells were incubated in a sterile incubator at 37°C with 5% CO_2_.

C57BL/6J mice were purchased from Saiye Bio‐pharmaceutical Co., Ltd (Guangdong, China). All animal experiments were approved by the Animal Ethics Committee of Guangzhou Meyers Biotechnology (NO. IACUC‐MIS2026021).

### 2.2 Synthesis of CTT/MPDA@L‐Ser NPs

Preparation of MPDA: MPDA nanoparticles were fabricated as described previously [30]. Briefly, 0.2 g F127 and 250 µL TMB were added to 50 mL ethanol and deionized water (V_ethanol_: V_deionized water_ = 1:1) and stirred for 30 min at 500 rpm. Then, 45 mg Tris was dissolved in 10 mL of water, after which 10 mL Tris buffer and 60 mg DA were added to the above mixture solution and continuously stirred for 24 h. After the reaction proceeded for 24 h, the nanoparticles were collected by centrifugation. The collected nanoparticles were then washed three times with an ethanol/acetone mixture (v/v = 2:1) via centrifugation, yielding the final MPDA NPs.

Loading of CTT: 2 mL CTT solution (2 mg/mL) was dropped into 2 mL MPDA nanoparticle solution (2 mg/mL), and the mixture was stirred for 1 h to load CTT. The encapsulation efficiency (EE) and drug loading (DL) of CTT were calculated using the following equation:

$$\begin{array}{ccc} \mathrm{EE}\;(\%) & = & (\text{Weight of encapsulated drug})/\\ & & (\text{Weight of total drug})\times 100\% \end{array}$$

$$\begin{array}{ccc} \mathrm{DL}\;(\%) & = & (\text{Weight of encapsulated drug})/\\ & & (\text{Weight of total nanoparticles})\times \;100\% \end{array}$$

Modification of L‐Ser on the CTT/MPDA NP surface: L‐Ser modification was performed according to a previously reported method [31]. Briefly, L‐Ser and CTT/MPDA NPs were separately dispersed in Tris solution (pH adjusted to 8.5). Subsequently, 2 mL of the L‐Ser solution was added to the CTT/MPDA NPs dispersion, and the mixture was stirred for 24 h.

### 2.3 Characterization of CTT/MPDA@L‐Ser NPs

The mean diameter, size distribution, and zeta potential of CTT/MPDA@L‐Ser NPs were measured using Zeta View (Zeta View PMX120, Particle Metrix, Germany). The particle morphology of CTT/MPDA@L‐Ser NPs were characterized using a scanning electron microscope (SEM, TESCAN MIRA LMS, Czech Republic). The elemental compositions of L‐Ser, MPDA, CTT/MPDA NPs, and CTT/MPDA@L‐Ser NPs were characterized by X‐ray photoelectron spectroscopy (XPS, Thermo Scientific K‐Alpha, USA). The chemical characteristics of L‐ser, MPDA, CTT/MPDA NPs, and CTT/MPDA@L‐Ser NPs were analyzed by Fourier‐transform infrared (FTIR, Thermo Fisher Scientific Nicolet iN10, USA). The specific surface area and pore size distributions of MPDA NPs were measured using a fully automated Brunauer–Emmett–Teller (BET) surface (volume) analyzer (Micromeritics ASAP 2460, USA). The stability of CTT/MPDA@L‐Ser NPs was evaluated by measuring particle size changes during storage at 4°C for 7 days.

In vitro drug release: 500 µL of CTT/MPDA@L‐Ser NPs at a concentration of 30 mg/mL was loaded into a dialysis bag (molecular weight cutoff, 3500 Da). The dialysis bag was then placed in dispersion media with pH 5.5, 7.4, and H_2_O_2_ (2 mmol/L), respectively, followed by incubation in a constant‐temperature shaker at 37°C with continuous oscillation. At predetermined time points, 1.5 mL of release solution was collected, and 1.5 mL of the corresponding fresh release medium was replenished simultaneously. The cumulative drug release rate was calculated by UV spectrophotometry.

### 2.4 In Vitro and in Vivo Safety Evaluation of Nanoparticles

Hemolysis test: 200 µL of RBC dilution was incubated with a series of MPDA and CTT/MPDA@L‐Ser NPs solution (1 mL) at concentrations ranging from 25 to 100 µg/mL. Triton X‐100 and PBS were used as positive (100% hemolysis) and negative (0% hemolysis) controls, respectively. After 4 h of incubation at 37°C, the samples were centrifuged at 3000 rpm for 15 min, and the absorbance of the supernatant was measured at 576 nm. The hemolysis rate was calculated as follows:

$$\begin{array}{ccc} \mathrm{Hemolysis}\;(\%) & = & (A_{576}\;\mathrm{of}\;\mathrm{sample}\;-\;A_{576}\;\mathrm{of}\;\mathrm{negative})/\\ & & (A_{576}\;\mathrm{of}\;\mathrm{positive}\;-\;A_{576}\;\mathrm{of}\;\mathrm{negative})\;\times \;100\% \end{array}$$

In vivo safety evaluation: To evaluate the short‐term and long‐term toxicity of nanoparticles in mice, C57BL/6J mice were injected with CTT/MPDA@L‐Ser NPs for 1, 7, and 14 days, respectively. The mice were euthanized at the corresponding time points, and the major organs (heart, liver, spleen, lung, and kidney) and serum were collected for H&E staining and blood parameter evaluation.

### 2.5 In Vitro Cellular Uptake and Cell Viability

HK‐2 cells were seeded in 24‐well plates at a density of 2 × 10^5^ cells per well and cultured for 24 h. Then, the cells were treated with different Coumarin 6 (C6)‐labeled nanoparticle solutions for 4 h. The experiment included four groups: (1) PBS + MPDA, (2) PBS + MPDA@L‐Ser, (3) DDP + MPDA, and (4) DDP + MPDA@L‐Ser. The concentration of DDP was 20 µM, and the nanoparticle concentration was 40 µg/mL. After a 4 h incubation period, the cells were washed twice with PBS and fixed with 4% paraformaldehyde for 10 min. Finally, the cells were visualized using confocal laser scanning microscopy (CLSM, LSM900, ZEISS).

HK‐2 cells were seeded in 96‐well plates at a density of 1 × 10^4^ cells per well and incubated for 24 h. The culture medium was then replaced with PBS, DDP, DDP+CTT, DDP+CTT/MPDA NPs (C/M), and DDP+ CTT/MPDA @L‐Ser NPs (C/M‐Ser), and the cells were incubated for 24 h. Then the CCK‐8 kit was used, and the absorbance of each well was determined by a microplate reader (ELx800, Biotek, USA) to determine the viability of cells.

### 2.6 In Vitro Antioxidant Effect of CTT/MPDA @L‐Ser

The generation of intracellular ROS was measured using the DCFH‐DA probe. HK‐2 cells were seeded in 24‐well plates at a density of 2 × 10^5^ cells per well and incubated for 24 h. The culture medium was then replaced with PBS, DDP, DDP+CTT, DDP+CTT/MPDA NPs (C/M), and DDP+ CTT/MPDA @L‐Ser NPs (C/M‐Ser), and the cells were incubated for 24 h. The nanoparticle concentration was 40 µg/mL. The administered dose of CTT was calculated based on the CTT content in CTT/MPDA@L‐Ser. After removing the medium, the DCFH‐DA probe was added to the cells and incubated for 20 min. The probe was subsequently replaced with PBS and observed by CLSM.

Glutathione (GSH) concentration in cells was measured using a glutathione assay kit. HK‐2 cells were seeded in 5 cm cell culture dishes and incubated for 24 h. After removing the medium, the cells were treated with PBS, DDP, DDP+CTT, DDP+C/M, and DDP+C/M‐Ser, and incubated for 24 h. Subsequently, the cells were collected, and the GSH level was measured according to the protocol provided with the glutathione assay kit. In brief, 0.5 mL of the cell sample was mixed thoroughly with 2 mL of reagent I working solution and centrifuged at 3500–4000 rpm for 10 min. Then, 1 mL of the supernatant was used for the chromogenic reaction. After standing for 5 min, the absorbance was measured at 420 nm.

### 2.7 Malondialdehyde (MDA) Level in Cells

The MDA concentration in cells was measured using an MDA assay kit. HK‐2 cells were seeded in 5 cm cell culture dishes and incubated for 24 h. After removing the medium, the cells were treated with PBS, DDP, DDP+CTT, DDP+C/M, and DDP+ C/M‐Ser, and incubated for 24 h. The MDA level was detected according to the protocol. In brief, the sample and reagents were added sequentially and incubated in a boiling water bath at a temperature above 95°C for 40 min. The sample was cooled under running water and centrifuged at 3500–4000 rpm for 10 min. The supernatant was collected, and the absorbance was measured at 532 nm.

### 2.8 Analysis of Mitochondrial Morphology and Membrane Potential in Cells

HK‐2 cells were seeded in 5 cm cell culture dishes and incubated for 24 h. After removing the medium, the cells were treated with PBS, DDP, DDP+CTT, DDP+C/M, and DDP+ C/M‐Ser, and incubated for 24 h. The cells were washed three times with PBS, and fixed with 2.5% glutaraldehyde solution. The fixed cells were prepared for biological transmission electron microscopy (Bio‐TEM) to observe mitochondrial morphology.

The mitochondrial membrane potential was detected using a JC‐1 mitochondrial membrane potential assay kit. HK‐2 cells were seeded in 24‐well plates at a density of 2 × 10^5^ cells per well and incubated for 24 h. The culture medium was then replaced with DDP, DDP+CTT, DDP+C/M, and DDP+ C/M‐Ser, and the cells were incubated for an additional 24 h. After removing the medium, the JC‐1 probe was added to the cells and incubated for 30 min. The probe was subsequently replaced with PBS and observed at 488 and 598 nm by CLSM.

### 2.9 Distribution of CTT/MPDA@L‐Ser In Vivo

C57BL/6J mice were administered cisplatin (20 mg/kg, i.p.) to establish an AKI model. On day 3 post‐cisplatin administration, mice were randomly allocated into four groups: (1) healthy mice treated with DiR/MPDA nanoparticles, (2) healthy mice treated with DiR/MPDA@L‐Ser nanoparticles, (3) AKI mice treated with DiR/MPDA nanoparticles, and (4) AKI mice treated with DiR/MPDA@L‐Ser nanoparticles. Fluorescence signals in major organs (heart, liver, spleen, lung, and kidney) were detected using an imaging system (Tanon imaging system, Tanon, China) at 4 and 24 h.

### 2.10 In Vivo Effect of CTT/MPDA@L‐Ser NPs on AKI Alleviation

An AKI model was established by intraperitoneal injection of DDP (20 mg/kg). The mice were randomly divided into five groups: (1) healthy mice injected with 0.9% saline, (2) AKI mice injected with 0.9% saline, (3) AKI mice injected with CTT drug solution, (4) AKI mice injected with C/M, and (5) AKI mice injected with C/M‐Ser (10 mg/kg). The mice were injected with drugs at 30 min before modeling and on days 1 and 2 after modeling. All mice were sacrificed 72 h after AKI model establishment, and mouse serum and kidneys were collected for further experiments.

### 2.11 Western Blotting

Immunoblotting analysis of proteins extracted from tissue lysates was performed. Frozen tissues were homogenized in lysis buffer prior to protein extraction. After centrifugation at 12000 rpm for 30 min at 4°C, the supernatants were collected, and total protein concentrations were quantified using a BCA Protein Assay Kit (Beyotime, Shanghai, China). Protein samples were then separated by SDS‐PAGE (12.5% gels) and transferred onto PVDF membranes (Merck Millipore, MA, USA). After blocking with 5% non‐fat milk, the membranes were incubated overnight at 4°C with the following primary antibodies: anti‐DPEP1 (1:2000), anti‐GPX4 (1:5000), anti‐SLC7A11 (1:1000), and anti‐Kim‐1 (1:1000). Bound antibodies were detected using horseradish peroxidase (HRP)‐conjugated IgG (Multi Sciences) and visualized with enhanced chemiluminescence (ECL) detection reagents (NCM Biotech, Suzhou, China). GAPDH and *β*‐actin served as loading controls.

### 2.12 Hematoxylin and Eosin (H&E) Staining and Prussian Blue Staining

The collected kidney tissues were fixed in 4% paraformaldehyde (PFA), embedded in paraffin, and sectioned at 3 µm thickness. Tubular injury was evaluated based on the following pathological features: luminal dilatation, epithelial necrosis, cast formation, and brush border loss. Histopathological changes were semiquantitatively scored on a 0–5 scale in a blinded manner according to the percentage of damaged tubules: 0 = normal, 1 = < 10%, 2 = 10%–25%, 3 = 26%–50%, 4 = 51%–75%, and 5 = > 75%. After dewaxing and rehydration of the paraffin sections following standard protocols, DAB‐enhanced Prussian blue staining was performed to detect iron deposition in the tissues (Solarbio, Beijing).

### 2.13 Immunofluorescence Staining and Immunohistochemistry

Kidney paraffin sections were permeabilized with immunostaining permeabilization buffer (Beyotime Biotechnology) according to the manufacturer's protocol. Sections were then incubated with primary antibodies, including rabbit anti‐DPEP1 or mouse anti‐Kim‐1, diluted in appropriate buffer. After washing, the sections were incubated with species‐specific fluorescent secondary antibodies, including Alexa Fluor 488‐conjugated goat anti‐rabbit IgG (Beyotime Biotechnology) or Alexa Fluor 555‐conjugated donkey anti‐mouse IgG (Beyotime Biotechnology), diluted in buffer. Nuclei were counterstained with 4’,6‐diamidino‐2‐phenylindole (DAPI). Fluorescence images were acquired using CLSM.

Paraffin sections were dewaxed and rehydrated and treated with citrate buffer (pH 6.0) in a 95°C water bath for 20 min. The sections were rinsed three times with PBS (pH 7.4), and then treated with 3% hydrogen peroxide for 10 min. Next, they were blocked with 5% goat serum for 30 min. After incubation with the NGAL primary antibody, the sections were incubated overnight at 4°C. Excess primary antibody was removed, and the sections were incubated with the secondary antibody for 1 h, followed by DAB staining. After staining, the sections were dehydrated, mounted, and observed under a microscope.

### 2.14 Statistical Analysis

All experiments were independently repeated at least three times (*n* ≥ 3), and all data were presented as the mean ± standard deviation (SD) of independent determinations. Statistical differences between two groups were analyzed using Student's t‐test, and differences among three or more groups were evaluated using one‐way analysis of variance (ANOVA) in GraphPad Prism 6. *P* < 0.05 was defined as statistically significant difference. *n.s*. indicates no statistically significant difference; ^*^ indicates *p* < 0.05; ^**^ indicates *p* < 0.01; ^***^ indicates *p* < 0.001; ^****^ indicates *p* < 0.0001.

## 3 Results

### 3.1 Characterization of CTT/MPDA@L‐Ser NPs

**FIGURE 2:**
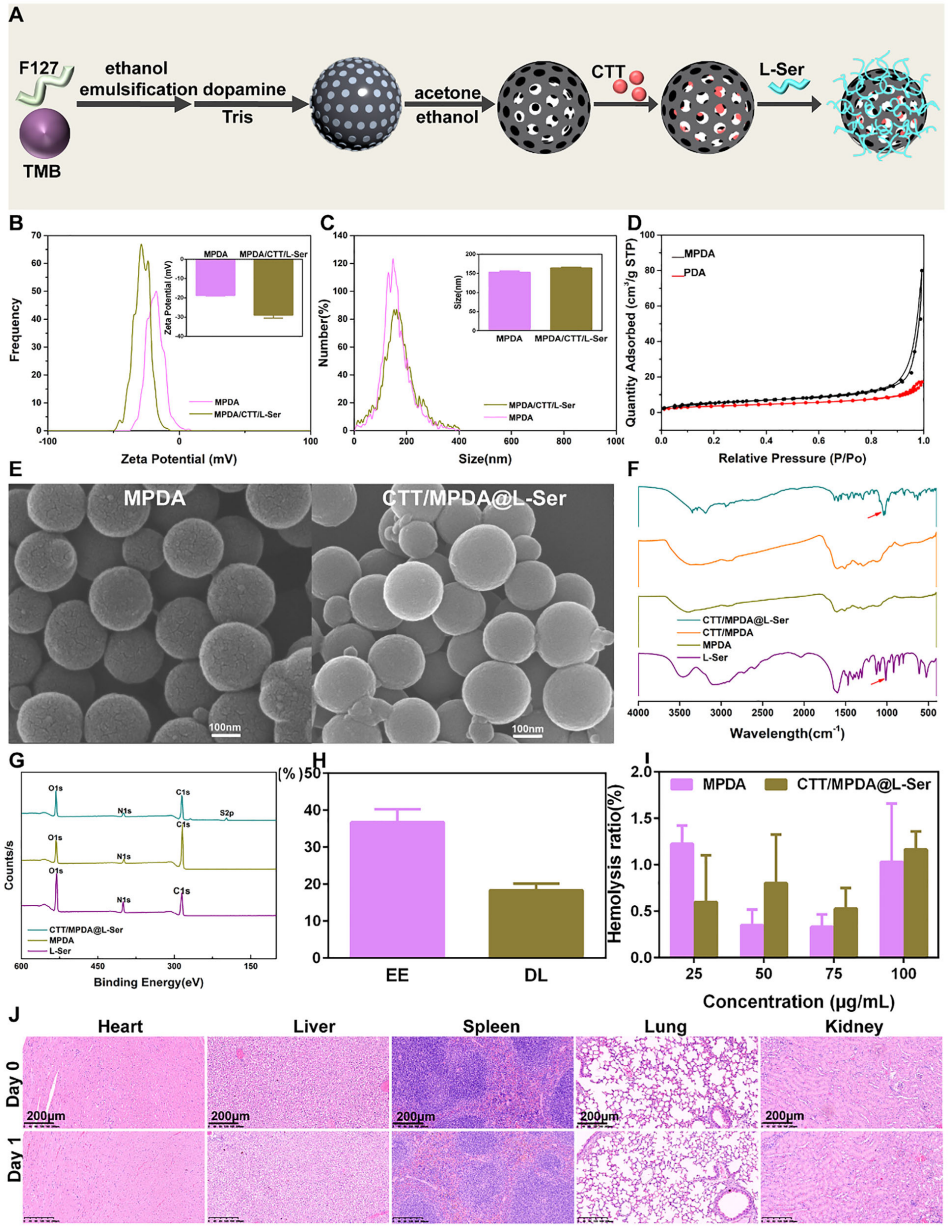
Characterization and in vitro and in vivo safety of nanoparticles. (A) Preparation of CTT/MPDA@L‐Ser NPs. (B) Zeta potential of MPDA NPs and CTT/MPDA@L‐Ser NPs. (C) Size distribution of MPDA NPs and CTT/MPDA@L‐Ser NPs. (D) N_2_ adsorption–desorption isotherms of PDA NPs and MPDA NPs. (E) SEM images of MPDA NPs (left) and CTT/MPDA@L‐Ser NPs (right). (F) FTIR spectra and (G) XPS spectra of different materials. (H) EE% and DL% of CTT/MPDA@L‐Ser NPs. (I) Hemolysis of MPDA NPs and CTT/MPDA@L‐Ser NPs (*n* = 3). (J) H&E sections of various organs after intravenous treatment with CTT/MPDA@L‐Ser NPs for 24 h.

Figure 2A illustrates the preparation process of CTT/MPDA@L‐Ser NPs. The average zeta potentials of MPDA NPs and CTT/MPDA@L‐Ser NPs were ‐18.73 ± 0.29 and ‐29.03 ± 1.25, respectively (Figure 2B). The average diameters of CTT/MPDA NPs and CTT/MPDA@L‐Ser NPs were 153.23 ± 2.54 and 164.03 ± 1.87 nm, respectively (Figure 2C). Moreover, the MPDA NPs showed a Brunauer–Emmett–Teller (BET) surface area of 20.57 m^2^/g (Figure 2D), which was larger than that of PDA NPs (nonporous) (13.04 m^2^/g), making them ideal for drug loading. The pore size of MPDA NPs was approximately 1.48 nm (Figure S1). SEM images confirmed the well‐defined spherical morphology and uniform size distribution of MPDA NPs and CTT/MPDA@L‐Ser NPs (Figure 2E). FTIR analysis of L‐Ser, MPDA, CTT/MPDA NPs, and CTT/MPDA @L‐Ser NPs were illustrated in Figure 2F. The CTT/MPDA@L‐Ser NPs exhibited an enhanced absorption band around 3400 cm^−^^1^, which can be attributed to overlapping stretching vibrations of hydroxyl and amino groups from the L‐Ser molecule. A peak emerged at 1740 cm^−^^1^, corresponding to the stretching vibration of the C═O group in the L‐Ser molecule. Moreover, a new peak emerged at 1038 cm^−^^1^, corresponding to the stretching vibration of the C─O and C─N groups in the L‐Ser molecule (The red arrow in Figure 2F). In this study, XPS analysis was primarily used to characterize the elemental composition of each nanoparticle group. As shown in Figure 2G, compared with the MPDA and the L‐Ser groups, the CTT/MPDA@L‐Ser NPs group contained C, N, O, and S elements, confirming the successful loading of CTT. Furthermore, the nitrogen content of CTT/MPDA @L‐Ser NPs was 7.36, which was much higher than that of MPDA (Table S1). Together, these results convincingly demonstrate the successful immobilization of L‐Ser. The EE% and DL% of CTT/MPDA@L‐Ser was 36.74% ± 3.52 and 18.37% ± 1.76, respectively (Figure 2H), demonstrating that MPDA nanoparticles represent a promising drug delivery carrier. The nanoparticles stored in different media showed no significant change in particle size over 7 days, indicating good dispersion stability (Figure S2). After 48 h of incubation in a medium at pH 7.4, the cumulative drug release from the nanoparticles was only 14.53%. In contrast, the cumulative release reached 66.50 and 62.60% in pH 5.5 medium and H_2_O_2_ solution, respectively. These results indicate that the MPDA‐based nanosystem likely possesses both pH and ROS‐responsive properties (Figure S3).

### 3.2 In Vitro and In Vivo Safety Evaluation of Nanoparticles

To further evaluate the blood biocompatibility of the nanoparticles, hemolysis assays were conducted. (Figure 2I and Figure S4). Even at a high concentration of 100 µg/mL (based on MPDA concentration), MPDA and MPDA@L‐Ser NPs did not exhibit significant hemolysis. The hemolysis rate was considerably lower than the 5% safety threshold, indicating negligible hemolytic potential and supporting the hemocompatibility of the nanoparticles for intravenous applications.

Hematological and histological analyses confirmed the systemic safety profile of the nanoparticles. Short‐term toxicity assessment showed that complete blood count (CBC) analysis revealed no significant alterations in red blood cells, leukocytes, platelets, or hemoglobin levels across treatment groups compared to controls (Figure S5), demonstrating favorable hemocompatibility. Furthermore, corresponding histological analysis of H&E‐stained sections from major organs, including the heart, kidney, liver, lung, and spleen, showed no signs of pathological changes such as inflammation, necrosis, or structural damage after nanoparticle treatment (Figure 2J). Similarly, no abnormalities were detected in routine blood tests (Table S2) or H&E‐stained pathological sections of mice (Figure S6) at 7 and 14 days after nanoparticle injection in vivo, indicating that the nanoparticles also exhibit favorable long‐term biosafety in vivo. These findings collectively indicate that the nanoparticle formulations exhibit good systemic biocompatibility for therapeutic applications.

### 3.3 Protective Mechanism of CTT/MPDA@L‐Ser in HK‐2 Cells

**FIGURE 3:**
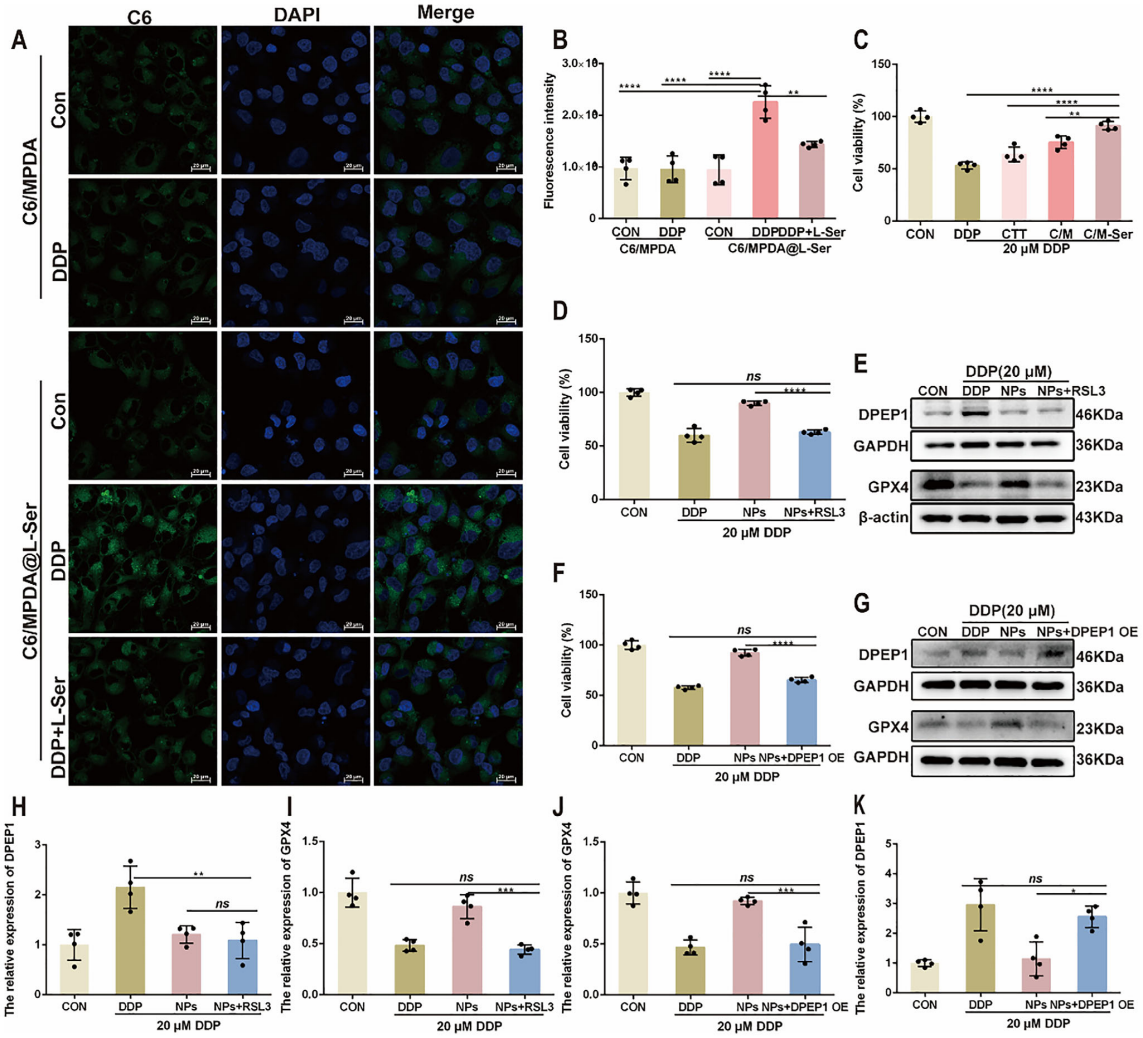
Protective mechanism of CTT/MPDA@L‐Ser in HK‐2 cells. (A) CLSM images and (B) flow cytometric quantification of nanoparticle cellular uptake. (C) Cell viability after different treatments. (D) Cell viability after treatment with CTT/MPDA@L‐Ser NPs or CTT/MPDA@L‐Ser NPs + RSL3. (E) Relative expression of GPX4 and DPEP1 proteins after treatment with CTT/MPDA@L‐Ser NPs or CTT/MPDA@L‐Ser NPs + RSL3. (F) Cell viability after treatment with CTT/MPDA@L‐Ser NPs or CTT/MPDA@L‐Ser NPs + DPEP1 overexpression. (G) Relative expression of GPX4 and DPEP1 proteins after treatment with CTT/MPDA@L‐Ser NPs or CTT/MPDA@L‐Ser NPs + DPEP1 overexpression. Semi‐quantitative analyses of (H) DPEP1, (I) GPX4, (J) GPX4, and (K) DPEP1 protein levels (*n* = 4).

Cellular uptake of nanoparticles in HK‐2 cells was evaluated by CLSM. After 4 h of incubation with the respective nanoparticle formulations, CLSM imaging revealed significantly enhanced fluorescence intensity in DDP+C6/MPDA@L‐Ser‐treated cells compared with other groups, indicating superior nanoparticle internalization (Figure 3A,B). Notably, after blocking with free L‐Ser, the uptake of C6/MPDA@L‐Ser NPs by DDP‐induced HK‐2 cells decreased significantly. This further indicates that the prepared MPDA@L‐Ser NPs more readily enter DDP‐induced HK‐2 cells, thereby facilitating drug release at the injury site. This enhanced uptake mechanism originates from cisplatin‐induced overexpression of KIM‐1 receptors on HK‐2 cell membranes (Figure S7). L‐Ser specifically targets these receptors, facilitating nanoparticle internalization. Cell viability in the different nanoparticle solution groups was higher than 85%, with no significant difference compared with the control group after 24 h of culture, indicating good compatibility of the nanoparticles (Figure S8). To screen the optimal administration concentration, DDP‐induced HK‐2 cells were treated with different drug concentrations. Figure S9D showed that when the nanoparticle concentration was 40 µg/mL (equivalent to 15 µg/mL CTT), a good cytoprotective effect was achieved. When the concentration was increased to 60 µg/mL (equivalent to 22 µg/mL CTT), the cell survival rate was higher than that at 40 µg/mL, but there was no significant difference. This might be because the cellular uptake capacity had reached saturation. Based on this, a concentration of 40 µg/mL CTT/MPDA@L‐Ser NPs was ultimately selected for subsequent cell experiments. When 40 µg/mL C/M‐Ser NPs were used to treat DDP‐induced HK‐2 cells, the cell survival rate reached 91.30%, which was significantly different from that after treatment with CTT or C/M NPs alone. This indicates that MPDA nanoparticles modified with L‐Ser exhibit higher drug delivery efficiency (Figure 3C).

To elucidate the molecular mechanism by which CTT/MPDA@L‐Ser NPs inhibited ferroptosis in AKI, we quantified key regulatory molecules within the ferroptosis signaling network. The accumulation of lipid peroxides is a hallmark feature of ferroptosis. GPX4 is a GSH‐dependent antioxidant enzyme that catalyzes the reduction of lipid peroxides, converting them into non‐toxic lipid alcohols and thereby suppressing lipid peroxidation in cell membranes. Thus, GPX4 serves as a central inhibitor of this regulated cell death pathway [32]. The cell cytotoxicity assay showed that treatment with CTT/MPDA@L‐Ser NPs significantly increased the viability of cisplatin‐induced HK‐2 cells. However, this protective effect was markedly attenuated upon co‐treatment with the GPX4 inhibitor RSL3 (Figure 3D). Subsequently, we further investigated protein expression profiles. Western blot analysis revealed that DDP stimulation upregulated DPEP1 expression while downregulating GPX4 in HK‐2 cells. Treatment with CTT/MPDA@L‐Ser NPs effectively reversed these alterations by decreasing DPEP1 levels and significantly increasing GPX4 expression. Notably, this GPX4 upregulation was abolished upon co‐treatment with the GPX4 inhibitor RSL3. These results collectively suggest that CTT/MPDA@L‐Ser NPs may attenuate ferroptosis by suppressing DPEP1 expression and subsequently activating the GPX4 pathway (Figure 3E,H,I). To further confirm that the nanoparticles exert their anti‐ferroptotic effect via the DPEP1‐mediated ferroptosis pathway, we constructed a DPEP1 overexpression plasmid for additional validation. Figure 3F shows that the cell survival rate in the CTT/MPDA@L‐Ser NPs group was 92.36%, whereas after DPEP1 overexpression and subsequent NPs treatment, the cell survival rate was only 65.37%. Similarly, Western blotting results (Figure 3G,J,K) demonstrate that DPEP1 overexpression followed by NPs treatment did not upregulate GPX4 protein expression in cells.

### 3.4 In Vitro Antioxidant Effect of CTT/MPDA@L‐Ser NPs

**FIGURE 4:**
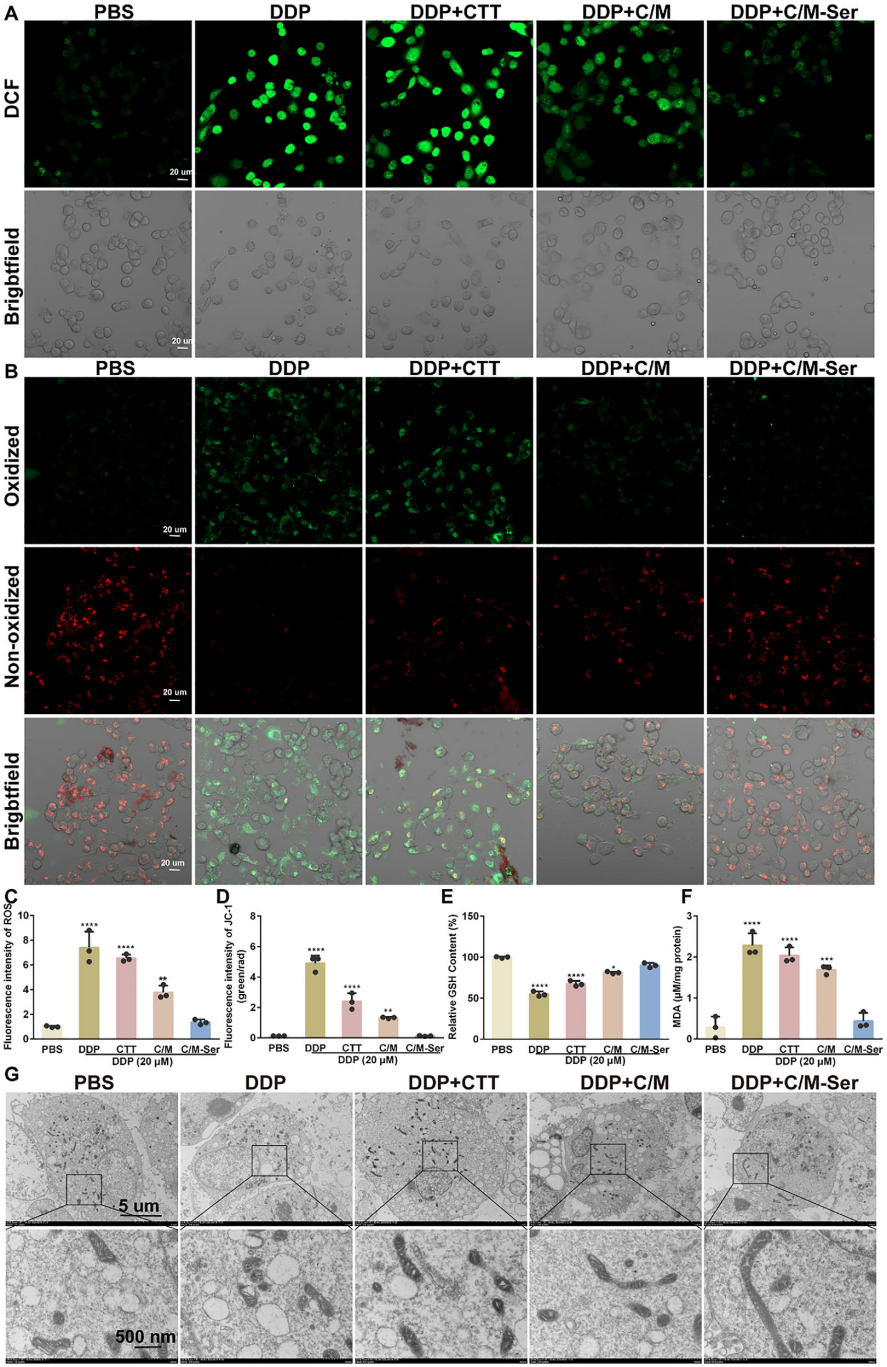
CTT/MPDA@L‐Ser NPs can reverse cisplatin‐induced ferroptosis in HK‐2 cells. (A) CLSM images of ROS in HK‐2 cells. (B) CLSM images with JC‐1 staining of HK‐2 cells. Semi‐quantitative analysis of (C) ROS and (D) mitochondrial membrane potential in HK‐2 cells after different treatments. (E) Relative GSH levels and (F) MDA in HK‐2 cells. Data are expressed as the mean ± SD,^*^ *p* < 0.05, ^**^ *p* < 0.01, ^***^ *p* < 0.001, ^****^ *p* < 0.0001 versus C/M‐Ser group (*n* = 3). (G) Bio‐TEM images of HK‐2 cells. The hallmark morphological changes of ferroptosis include mitochondrial shrinkage, increased membrane density, disappearance of cristae, and outer membrane rupture.

Intracellular ROS oxidize DCFH‐DA to fluorescent DCF (green emission), therefore, the fluorescence intensity of DCF can be used to quantitatively reflect intracellular ROS levels. As shown in Figure 4A,C, treatment with DDP induced a substantial increase in intracellular ROS levels, which were approximately 7‐fold higher than those in the control group. ROS levels in the CTT and C/M groups were approximately 6‐ and 4‐fold higher than those in the PBS group, respectively. In contrast, the C/M‐Ser group exhibited the lowest ROS signal, which was comparable to that of the control group. This enhanced efficacy is attributed to (i) L‐Ser‐mediated improvement in cellular internalization and (ii) the intrinsic ROS‐scavenging capacity of MPDA nanoparticles, which collectively reduce oxidative stress.

### 3.5 Anti‐ferroptosis Effect In Vitro

In this study, we also measured mitochondrial membrane potential using the JC‐1 fluorescent probe. When mitochondrial membrane potential is high, JC‐1 accumulates in the mitochondrial matrix as aggregates, emitting red fluorescence. Conversely, when mitochondrial membrane potential is low, JC‐1 exists in monomeric form, emitting green fluorescence. The relative ratio of red to green fluorescence can be used to assess the degree of mitochondrial depolarization and thereby evaluate mitochondrial damage. The decrease in mitochondrial membrane potential was represented by the ratio of green to red fluorescence intensity. As shown in Figure 4B,D, the fluorescence ratio in the PBS group was approximately 0.1. Following DDP treatment, the ratio increased to approximately 5, indicating a significant loss of mitochondrial membrane potential. In contrast, the ratios after CTT and C/M treatments were approximately 2 and 1, respectively. Notably, the C/M‐Ser group exhibited a fluorescence ratio of 0.1, which was not significantly different from that of the PBS group but was significantly lower than those observed in the CTT and C/M groups. These results indicated that CTT/MPDA@L‐Ser NPs treatment restored mitochondrial membrane potential and attenuated mitochondrial damage.

GSH is the main antioxidant in cells, and GSH depletion leads to inactivation of the GPX4 enzyme, which causes intracellular lipid peroxidation accumulation and induces ferroptosis [33]. As shown in Figure 4E, after DDP induced HK‐2 cells, the intracellular GSH level decreased to 55% of that in the control group. After treatment with C/M NPs, the intracellular GSH level was 81% of that in the control group. After treatment with C/M‐Ser, the cellular GSH level was 90% of that in the control group.

MDA is a key product of lipid peroxidation in ferroptosis, and its level reflects the degree of oxidative stress and cell damage, making it an important biomarker in ferroptosis research. After cisplatin stimulated HK‐2 cells, the intracellular MDA level significantly increased to 2.28 µM/mg. Treatment with C/M‐Ser significantly reduced the intracellular MDA level to 0.44 µM/mg (Figure 4F). These results indicate that CTT/MPDA/@L‐Ser NPs can effectively reduce intracellular peroxide accumulation and ferroptosis.

The hallmark morphological changes of ferroptosis include mitochondrial shrinkage, increased membrane density, disappearance of cristae, and outer membrane rupture. Therefore, we used Bio‐TEM to observe mitochondrial morphological changes in cells after different treatments, to confirm the occurrence of ferroptosis and demonstrate that our nanomedicine can mitigate ferroptosis in cells. As shown in Figure 4G, compared with the control group, mitochondria in the DDP group exhibited significant shrinkage, with most cristae disappearing. In the DDP + CTT group, mitochondria showed obvious shrinkage, partial disappearance of cristae, and some mitochondrial membrane rupture. In contrast, after treatment with C/M or C/M‐Ser, mitochondrial cristae were significantly restored, and mitochondrial morphology recovered.

To further verify that NPs protect DDP‐induced HK‐2 cells primarily through an anti‐ferroptotic effect, we additionally introduced the ferroptosis inhibitor Fer‐1 and the ferroptosis inducer RSL3 for comparative experiments. As shown in Figure S10A, after Fer‐1 treatment, the cell survival rate increased to 82.66%, and after treatment with CTT/MPDA@L‐Ser, the cell survival rate was 91.20%. There was no significant difference in cell survival between these two groups. In the Fer‐1 + CTT/MPDA@L‐Ser group, the cell survival rate was 96.78%. Subsequently, we induced ferroptosis in HK‐2 cells by stimulation with the ferroptosis inducer RSL3. After treatment with CTT/MPDA@L‐Ser NPs, the cell survival rate reached 97.12%, further demonstrating that CTT/MPDA@L‐Ser NPs possess excellent anti‐ferroptotic activity (Figure S10B). We further investigated the lipid peroxidation (LPO) inhibitory effects of Fer‐1 and NPs. As shown in Figure S11A,B, the LPO inhibition efficiency of NPs was comparable to that of Fer‐1, and no significant difference was observed between Fer‐1 + NP combination treatment and single NP treatment. Similarly, GPX4 expression (Figure S11C,D) was enhanced after treatment with Fer‐1, NPs, and Fer‐1 + NPs, and Fer‐1 + NPs did not show a more significant effect than the single‐agent treatments of Fer‐1 or NPs. Based on these results, we further verified that CTT/MPDA@L‐Ser NPs exert cytoprotective effects primarily by inhibiting ferroptosis.

### 3.6 Distribution of CTT/MPDA@L‐Ser NPs In Vivo

**FIGURE 5:**
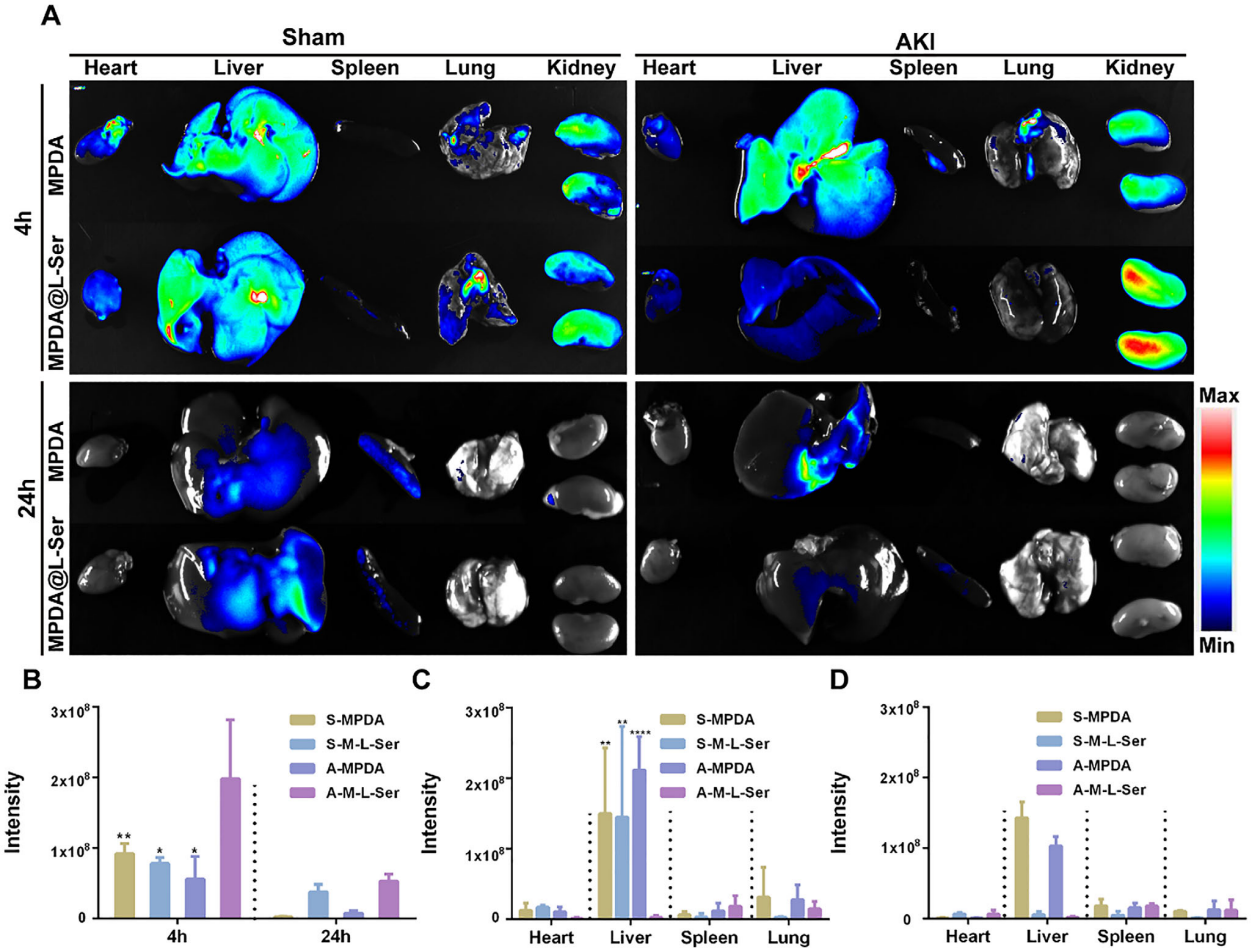
Distribution in major organs and renal accumulation of MPDA NPs and MPDA@L‐Ser NPs. S‐MPDA: sham group injected with MPDA NPs. S‐M‐L‐Ser: sham group injected with MPDA@L‐Ser NPs. A‐MPDA: AKI group injected with MPDA NPs. A‐M‐L‐Ser: AKI group injected with MPDA@L‐Ser NPs. (A) Fluorescence images of the major organs (heart, liver, spleen, lung, and kidney) of sham and AKI mice at 4 and 24 h after intravenous injection of MPDA and MPDA@L‐Ser. The nanoparticles were labeled with DiR; (B) Quantitative analysis of renal fluorescence intensity at 4 and 24 h after injection; (C, D) Quantitative analysis of fluorescence intensity in the heart, liver, spleen, and lung at 4 h and 24 h after injection, respectively. Data are expressed as the mean ± SD, ^*^ *p* < 0.05, ^**^ *p* < 0.01, ^***^ *p* < 0.001 versus the A‐M‐L‐Ser group (*n* = 3).

Kim‐1 was highly expressed on the surface of renal tubules during AKI [34]. In this study, Kim‐1 protein expression was markedly increased after DDP injection (Figure S12). Biodistribution analysis at 4 h post‐injection showed distinct nanoparticle accumulation patterns across organs (Figure 5A). In the sham group, nanoparticles were distributed in both the kidneys and liver, with higher accumulation observed in the liver. Following MPDA injection in the AKI group, nanoparticles also accumulated in both kidneys and liver, again demonstrating significantly higher hepatic accumulation. As anticipated, in the MPDA@L‐Ser‐treated AKI group, nanoparticles accumulated almost exclusively within the kidneys (Figure 5B,C). The accumulation efficiency of CTT/MPDA@L‐Ser NPs in healthy mice was lower than that in AKI‐induced mice. This demonstrates that targeting Kim‐1, which is highly expressed in AKI, using L‐Ser is feasible. At 24 h post‐injection, fluorescence signals from the nanoparticles were barely detectable in major organs, which may be attributed to their clearance from the body. Based on this clearance profile, a once‐daily dosing regimen was selected for subsequent experiments (Figure 5D). Collectively, surface modification of nanoparticles with L‐Serine significantly enhanced renal‐specific accumulation during AKI.

### 3.7 Effect of Alleviating AKI In Vivo

**FIGURE 6:**
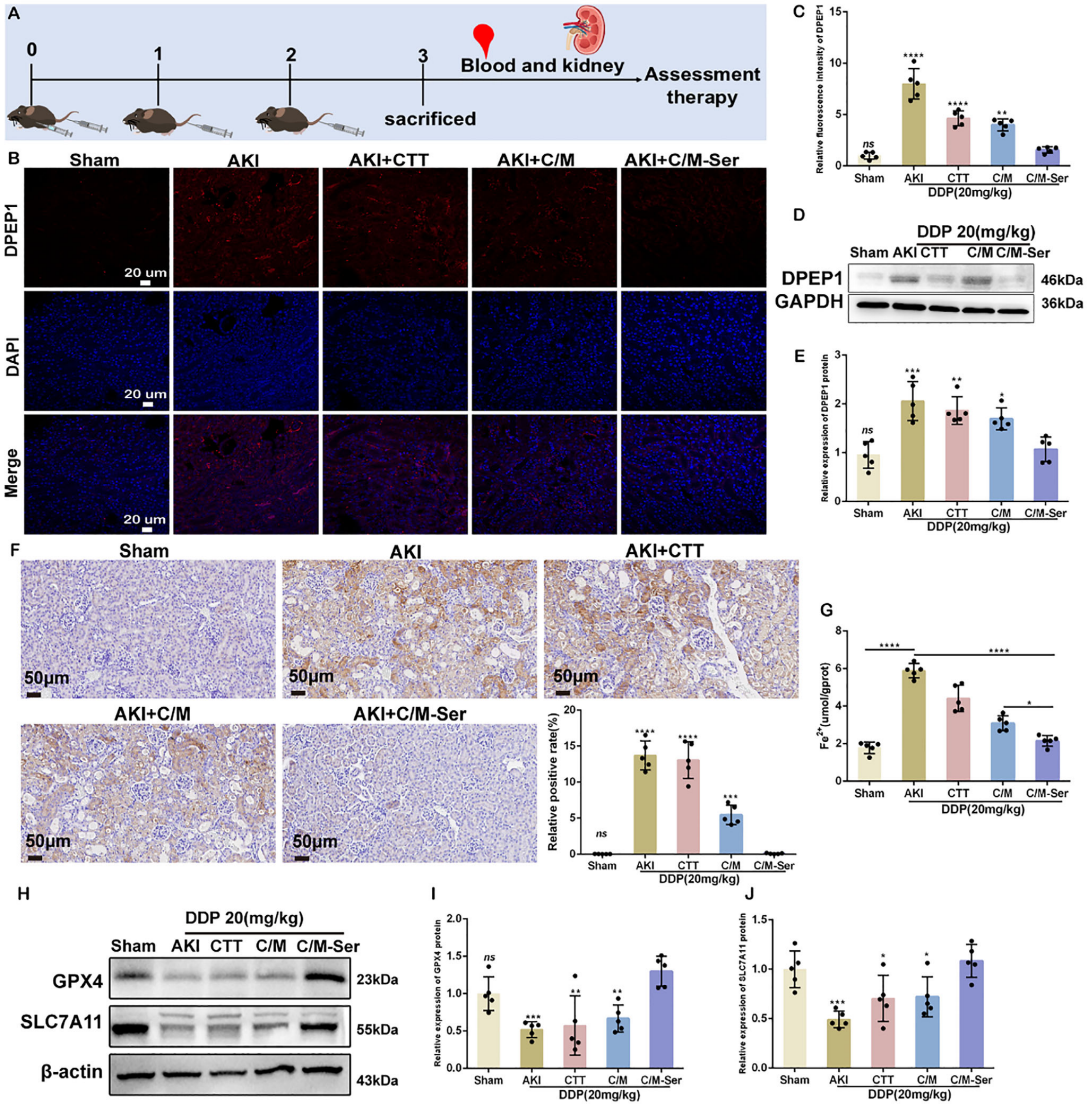
CTT/MPDA@L‐Ser NPs alleviate cisplatin‐induced disorder of iron metabolism in mouse kidneys. (A) Schematic diagram of the therapeutic regimen for DDP‐induced AKI. (B) DPEP1 immunofluorescence section image; (C) Immunofluorescence semi‐quantitative analysis of DPEP1; (D, E) Western blotting and semi‐quantitative analysis of DPEP1 protein; (F) Detection of iron ion deposition in kidney sections by DAB‐enhanced Prussian blue staining, and semi‐quantitative analysis of Prussian blue staining; (G) Tissue Fe^2+^ levels after different treatments; (H–J) Western blotting and semi‐quantitative analysis of GPX4 and SLC7A11 protein. Data are expressed as the mean ± SD, *ns*, not significant, ^*^ *p* < 0.05, ^**^ *p* < 0.01, ^***^ *p* < 0.001, ^****^ *p* < 0.0001 versus C/M‐Ser group (*n* = 5).

Figure 6A illustrates the experimental timeline and treatment regimen. Following sacrifice, plasma and kidney tissues were collected for analysis. Figure 6B,C present representative immunofluorescence staining images for DPEP1. Compared with the Sham group, kidney sections from AKI mice exhibited a marked increase in DPEP1 signal intensity (red fluorescence). Treatment with CTT, C/M, or C/M‐Ser resulted in varying degrees of attenuation of this DPEP1 fluorescence. Notably, the signal intensity in the C/M‐Ser group was the weakest, showing no significant difference compared with the Sham group. Furthermore, a significant difference in DPEP1 fluorescence intensity was observed between the C/M and C/M‐Ser groups. Consistent with this, DPEP1 protein expression was significantly upregulated in AKI kidneys but downregulated following C/M‐Ser NP administration (Figure 6D,E). These findings suggest that C/M‐Ser NPs exhibit enhanced renal‐targeting efficiency in AKI mice. Ferroptosis is characterized by intracellular iron ion accumulation; therefore, Prussian blue staining was performed to observe iron ion deposition in tissue sections (Figure 6F). The positive staining rates of the AKI, CTT, C/M, and C/M‐Ser groups were 13.72%, 13.07%, 5.46%, and 0.098% respectively. Furthermore, we quantitatively detected the Fe^2^^+^ content in the tissues. As shown in Figure 6G, the Fe^2^^+^ contents in the Sham, AKI, CTT, C/M, and C/M‐Ser groups were 1.79, 5.89, 4.42, 3.09, and 2.15 µmoL/gprot, respectively. The results demonstrate that the Fe^2^^+^ concentration in renal tissue decreased after C/M‐Ser treatment. Expression of the ferroptosis regulators GPX4 and SLC7A11 (critical for cystine uptake, GSH synthesis, and lipid peroxide clearance) (Figure 6H,I,J) was significantly suppressed in the AKI group, whereas C/M‐Ser NP treatment significantly restored SLC7A11 and GPX4 protein levels compared with both the AKI and CTT groups. This restoration indicates reduced lipid peroxidation and ferroptosis suppression. Furthermore, the levels of SLC7A11 and GPX4 were significantly higher in the C/M‐Ser NP group than in the C/M NP group, demonstrating that L‐Ser surface modification enhances renal accumulation and therapeutic efficacy against ferroptosis.

**FIGURE 7:**
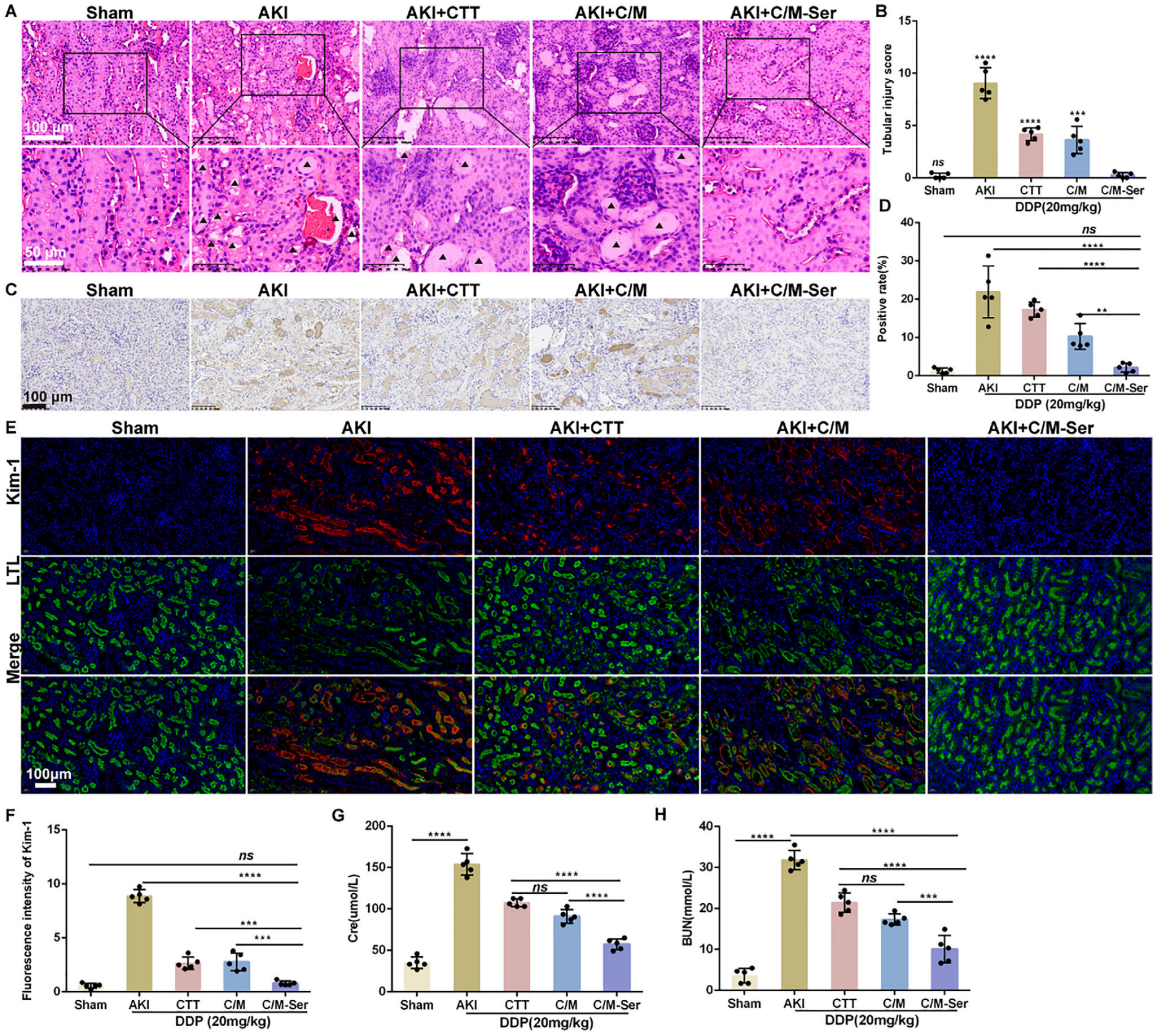
CTT/MPDA@L‐Ser NPs alleviate cisplatin‐induced acute kidney injury. (A) Representative H&E sections of the kidney in each group. Triangles indicate tubules with necrosis, epithelial anoikis cavitation, brush border loss, or cast formation; (B) Tubular injury scores were calculated according to the percentage of damaged tubules; (C) NGAL immunohistochemical sections images; (D) Semi‐quantitative analysis of NGAL by immunohistochemistry; (E) Kim‐1 immunofluorescence section images; (F) Immunofluorescence semi‐quantitative analysis of Kim‐1; (G, H) Levels of serum creatinine (Scr) and BUN after different treatments. Data are expressed as the mean ± SD, *ns*, not significant, ^*^ *p* < 0.05, ^**^ *p* < 0.01, ^***^ *p* < 0.001, ^****^ *p* < 0.0001 versus the C/M‐Ser group (*n* = 5).

Renal tissue sections were stained with hematoxylin and eosin (H&E) and evaluated by light microscopy to assess histological injury. Representative images and injury scores were shown in Figure 7A,B respectively. The results revealed preserved renal architecture with distinct proximal tubular brush borders in the sham group. Conversely, kidneys from AKI mice exhibited severe tubular damage, including extensive tubular necrosis, epithelial cell sloughing, brush border loss, and abundant hyaline casts. Treatment with various CTT formulations progressively attenuated these histopathological alterations, indicating renal reparative effects. Notably, the C/M‐Ser NPs group demonstrated near‐complete restoration of tubular architecture, comparable to that of the sham group. Tubular injury was quantified using a validated semi‐quantitative scoring system (0–20 scale) based on four parameters: tubular necrosis, epithelial sloughing, brush border loss, and hyaline cast formation. Severity grades were: 0 (normal), 1 (< 10%), 2 (10%–25%), 3 (26%–50%), 4 (51%–75%), and 5 (> 75% involvement). The injury scores were significantly elevated in the AKI group (9.06 ± 1.47) versus sham (0.18 ± 0.47). Treatment reduced scores to 4.18 ± 0.62 (CTT), 2.98 ± 0.41 (C/M NPs), and 0.24 ± 0.26 (C/M‐Ser NPs). The C/M‐Ser NPs group showed significantly lower injury scores than both the CTT and C/M NPs groups, confirming superior protective efficacy. The results of NGAL immunohistochemical staining (Figure 7C) and semi‐quantitative analysis (Figure 7D) showed that NGAL expression was upregulated in the kidneys of AKI mice, with a positive rate of 21.94%. The positive rates in the CTT, C/M NPs and C/M@L‐Ser NPs groups were 17.28%, 10.25% and 2.12%, respectively, demonstrating that C/M@L‐Ser NPs exert excellent renal protective effects. Figure 7E,F presents representative immunofluorescence staining images for Kim‐1. Compared with the Sham group, kidney sections from AKI mice exhibited a marked increase in Kim‐1 signal intensity (red fluorescence). Treatment with CTT, C/M, or C/M‐Ser resulted in varying degrees of attenuation of Kim‐1 fluorescence. Notably, the signal intensity in the C/M‐Ser group was the weakest, showing no significant difference compared with the Sham group. Furthermore, a significant difference in Kim‐1 fluorescence intensity was observed between the C/M and C/M‐Ser groups. These findings suggest that C/M‐Ser NPs exhibit enhanced renal‐targeting efficiency in AKI mice. To further assess renal functional recovery, serum creatinine (Scr) and blood urea nitrogen (BUN) levels were determined in mice (Figure 7G,H). Scr and BUN levels were significantly elevated in AKI mice compared with the sham group. Treatment with C/M‐Ser nanoparticles (NPs) significantly reduced both Scr and BUN levels to values comparable to those of the sham group, indicating effective mitigation of AKI.

## 4 Discussion

CI‐AKI is a common dose‐dependent complication in treated patients, which not only reduces the effect of chemotherapy but also imposes an economic burden on patients. Clinical treatment of CI‐AKI involves mainly supportive therapy, but the therapeutic effect is poor, and the development of specific therapies is urgent. Ferroptosis has emerged as a major contributor to AKI because injured renal tubules are highly exposed to oxidative stress, iron dysregulation, and lipid peroxide accumulation. Therefore, we prepared CTT/MPDA@L‐Ser NPs for treating CI‐AKI. CTT is a commonly used antibacterial synergist in clinical practice with clear safety, and previous studies have shown that CTT can alleviate kidney disease and inhibit ferroptosis in an AKI model. MPDA not only has good biological safety, easy surface modification, and good drug loading ability but can also remove excessive amounts of ROS from cells. The Kim‐1 receptor is highly expressed on the kidney surface during AKI. Therefore, L‐Ser was modified on the surface of the MPDA NPs to help the nanoparticles target the kidney. The experimental results demonstrate that CTT/MPDA@L‐Ser NPs exhibit favorable biocompatibility.

Ferroptosis has been increasingly recognized as a central form of regulated tubular cell death in AKI. Ni et al. summarized that ferroptosis in AKI is characterized by iron accumulation, lipid peroxidation, mitochondrial damage, and impairment of the GPX4‐dependent antioxidant defense system [35]. In cisplatin‐induced AKI, Ikeda et al. further demonstrated that cisplatin promotes renal dysfunction together with increased ferroptosis‐related markers, whereas ferroptosis inhibition by Fer‐1 alleviates tubular injury [36]. Consistent with these studies, our data showed that cisplatin induced Fe^2^^+^ accumulation, increased MDA production, depleted GSH, reduced GPX4 and SLC7A11 expression, and caused typical ferroptotic mitochondrial injury in HK‐2 cells and AKI kidneys. These findings indicate that the therapeutic effect of CTT/MPDA@L‐Ser NPs is closely associated with the suppression of ferroptotic tubular injury rather than simple improvement of renal function alone.

Recent nanomedicine studies have attempted to alleviate AKI by targeting ferroptosis. Zhang et al. reported that anti‐ferroptotic nanotherapeutics could reduce lipid peroxidation and protect against AKI through modulation of ferroptosis‐related pathways [14]. More recently, Zeng et al. developed L‐serine‐modified ultrasmall nanodots with dual anti‐ferroptotic activity for cisplatin‐induced AKI, demonstrating the feasibility of combining injured renal tubule targeting with ferroptosis inhibition [37]. Compared with these approaches, the incremental contribution of our study lies in the integration of three functionally complementary components into one nanoplatform. First, L‐Ser modification enhanced the accumulation of nanoparticles in KIM‐1‐positive injured renal tubular cells. Second, MPDA served as a biocompatible carrier that improved cilastatin delivery and provided a favorable intracellular protective microenvironment. Third, cilastatin, a clinically used DPEP1 inhibitor, was repositioned to regulate DPEP1‐associated ferroptosis signaling. Guan et al. previously identified DPEP1/CHMP1A as a genetic locus involved in the regulation of ferroptosis and kidney disease progression [21]. In the present study, DPEP1 overexpression weakened nanoparticle‐mediated GPX4 restoration and cytoprotection, while RSL3 attenuated the GPX4‐dependent protective effect, further supporting the involvement of the DPEP1‐GPX4/SLC7A11 axis in the anti‐ferroptotic activity of CTT/MPDA@L‐Ser NPs. Therefore, this work extends current AKI ferroptosis‐targeted therapy by linking KIM‐1‐mediated injured tubular delivery with cilastatin‐based DPEP1 regulation, providing a targeted nanotherapeutic strategy for cisplatin‐induced AKI.

The present data support that the superior renoprotective effect of CTT/MPDA@L‐Ser NPs mainly arises from targeted delivery‐amplified regulation of DPEP1‐associated ferroptosis. In cisplatin‐injured HK‐2 cells and AKI kidneys, KIM‐1 was markedly upregulated, which enabled L‐Ser‐modified nanoparticles to accumulate preferentially in injured tubular cells. This targeting effect increased the local availability of cilastatin and explains why CTT/MPDA@L‐Ser NPs showed stronger protective efficacy than free CTT or non‐targeted CTT/MPDA NPs. After intracellular delivery, cilastatin‐loaded nanoparticles reduced DPEP1 expression and restored the ferroptosis‐suppressive proteins GPX4 and SLC7A11. Reduction in MDA production, Fe^2^^+^ deposition, mitochondrial membrane potential loss, and ferroptotic mitochondrial injury was also observed.

The rescue experiments further strengthened this interpretation. Pharmacological inhibition of GPX4 by RSL3 weakened the protective effect of CTT/MPDA@L‐Ser NPs, indicating that GPX4 restoration is required for the anti‐ferroptotic activity of the nanoplatform. Conversely, DPEP1 overexpression attenuated nanoparticle‐mediated GPX4 recovery and reduced cell survival, supporting the involvement of DPEP1 in the regulation of ferroptosis under cisplatin‐induced injury. Therefore, CTT/MPDA@L‐Ser NPs do not act simply as a general antioxidant system; instead, they function as a targeted cilastatin delivery platform that suppresses DPEP1‐associated ferroptotic signaling and restores the GPX4/SLC7A11‐dependent anti‐ferroptosis network. The ROS‐scavenging property of MPDA may further improve the intracellular oxidative microenvironment, but the major therapeutic advantage of this nanoplatform is its ability to concentrate cilastatin in injured renal tubules and thereby enhance DPEP1‐related ferroptosis inhibition. In addition to DPEP1‐associated ferroptosis regulation, cilastatin may also exert proximal tubule‐protective effects through megalin‐related mechanisms. Hori et al. reported that cilastatin can interfere with megalin‐mediated binding and uptake of cisplatin, thereby reducing cisplatin‐induced proximal tubular injury [38], and Matsushita et al. further showed that cilastatin ameliorates AKI through megalin interference [39]. Although megalin signaling was not directly examined in the present study, these findings provide an additional pharmacological background for cilastatin‐mediated nephroprotection and warrant further investigation in future studies.

Targeted delivery is another important determinant of therapeutic efficacy. Free cilastatin is rapidly handled by the kidney and is largely excreted in urine, which supports renal exposure but does not ensure preferential retention in injured tubular cells. In contrast, the L‐serine‐modified nanoplatform showed enhanced accumulation in AKI kidneys and reduced uptake after free L‐serine competition in vitro, supporting an active targeting component. Importantly, KIM‐1 is upregulated early after tubular injury and persists during the progression phase of AKI; therefore, targeting KIM‐1‐positive cells is still therapeutically meaningful even though ferroptosis begins early. The repeated administration schedule used here was designed to cover both the initiation and propagation phases of cisplatin‐induced tubular damage.

In summary, this study demonstrated that CTT/MPDA@L‐Ser NPs inhibited ferroptosis by suppressing DPEP1 expression and subsequently activating the GPX4 pathway. In vivo pharmacodynamic evaluations confirmed that ferroptosis inhibition by CTT/MPDA@L‐Ser NPs contributes to renal functional recovery in DDP‐induced AKI, highlighting the potential therapeutic value of this nanoplatform for clinical application.

## 5 Conclusion

In this study, we developed an MPDA nanodrug system loaded with CTT for treating cisplatin‐induced AKI. This research addressed three key issues: (1) targeted delivery of drugs to injured kidneys to enhance local drug accumulation; (2) therapeutic repositioning of CTT, which not only leverages its known safety profile but also opens new avenues for clinical management of AKI; (3) preliminary exploration into the mechanism by which CTT alleviates AKI by inhibiting ferroptosis. Specifically, CTT suppresses DPEP1, thereby upregulating the expression of downstream ferroptosis‐related proteins, including GPX4 and SLC7A11, and ultimately exerting an anti‐ferroptotic effect. Future investigations should systematically elucidate additional regulatory mechanisms of DPEP1 in AKI pathogenesis, thereby providing a robust scientific foundation for the clinical translation of cilastatin‐based nanotherapeutics in AKI treatment.

## Author Contributions

Qinghua Liu, Junbing He, Wanbing Qin, and Weiding Wang conceived and designed the experiments. Wanbing Qin, Jiaqi Huang, Manting Zhang, Wanchun Yang, Chi Jiang, Jiekai Li, Jinjuan Wen, Qibin Huang, and Shiqi Wen performed the experiments. Wanbing Qin and Jiaqi Huang analyzed the data. Wanbing Qin wrote the manuscript. Junbing He, Qinghua Liu, Weidong Wang, and Mingwei Xu revised the manuscript. All authors have given approval to the final version of the manuscript.

## Funding

This work was supported by the National Natural Science Foundation of China (82302446, 82470811), China foundation for youth entrepreneurship and employment (Grant No. P24052087757), Youth Top‐notch Talent of Guangdong TeZhi Plan (2024TQ08A155), GuangDong Basic and Applied Basic Research Foundation (2024A1515012890, 2025A1515010689, 2026A1515012073), Youth Scientific Research Project of Jieyang People's Hospital (ZX202303).

## Ethics Statement

The animal experimental protocols were reviewed and approved by the Animal Ethics Committee of Guangzhou Meyers Biotechnology (NO. IACUC‐MIS2026021), and were performed according to the National Institutes of Health guidelines for the care and use of laboratory animals.

## Conflicts of Interest

The authors declare no conflicts of interest.

## Supporting information




**Supporting File**: advs77090‐sup‐0001‐SuppMat.docx.

## Data Availability

The data that support the findings of this study are available on request from the corresponding author. The data are not publicly available due to privacy or ethical restrictions.
