# Supplementary material for: Kidney‐Targeted Cilastatin Nanoparticles Inhibit DPEP1‐Mediated Ferroptosis for Acute Kidney Injury Therapy
Source: Adv Sci (Weinh). 2026 Aug 13:e77090. Online ahead of print. doi: 10.1002/advs.77090 (PMC13472103; doi:10.1002/advs.77090)
Supplement: Supplementary file 1 [file ADVS-9999-e77090-s001.docx]

**Title:** **Kidney-Targeted Cilastatin Nanoparticles Inhibit DPEP1-Mediated Ferroptosis for Acute Kidney Injury Therapy**

**Author:**

Wanbing Qin^1^, Jiaqi Huang^1^, Manting Zhang^1^, Wanchun Yang^1^, Mingwei Xu^1^, Chi Jiang^1^, Jiekai Li^1^, Jinjuan Wen^1^, Qibin Huang^1^, Shiqi Wen^1^, Weidong Wang^1,2^**^*^**, Junbing He^1^**^*^**, Qinghua Liu^3,4,5^**^*^**

**Author details:**

1 Jieyang Medical Research Center, Jieyang People's Hospital, Jieyang, Guangdong, China

2 Department of Pathophysiology, Zhongshan School of Medicine, Sun Yat-sen University, Guangzhou, China

3 Department of Nephrology, The First Affiliated Hospital, Sun Yat-sen University, Guangzhou, Guangdong, China

4 Department of Nephrology, Jieyang People's Hospital, Jieyang, Guangdong, China

5 Guangdong Provincial Key Laboratory of Nephrology, NHC Key Laboratory of Clinical Nephrology (Sun Yat-sen University), Guangzhou, China

*** Correspondence:**

Qinghua Liu: liuqhua6@mail.sysu.edu.cn

Junbing He: [junbingg@gdmu.edu.cn](mailto:junbingg@gdmu.edu.cn)

Weidong Wang: wangwd6@mail.sysu.edu.cn

Fig.S1: Pore size distribution of MPDA NPs.

Table S1: Relevant elements percentage.

| Sample | C (%) | O (%) | N (%) | C/O | C/N |
| --- | --- | --- | --- | --- | --- |
| L-Ser | 53.20 | 34.11 | 12.55 | 1.56 | 4.24 |
| MPDA | 76.56 | 18.00 | 5.23 | 4.25 | 14.64 |
| CTT/MPDA | 75.30 | 18.78 | 5.73 | 4.01 | 13.14 |
| CTT/MPDA@L-Ser | 69.89 | 22.60 | 7.36 | 3.09 | 9.50 |

Fig.S2 Stability of the CTT/MPDA@L-Ser NPs at different time intervals (*n* = 3).

Fig S3: CTT release profile from CTT/MPDA@L-Ser NPs suspension release media at pH 5.5, pH 7.4 and H₂O₂（2 mmol/L）(*n* = 3).

Fig.S4: Nanoparticle hemolysis assay.

Fig.S5: Changes in blood routine examination parameters in various treatments after treating with CTT/MPDA@L-Ser for 24 h. Values are expressed as mean ± SD, (*n* = 3).

Table S2: Changes in blood routine examination parameters in various treatments. Values are expressed as mean ± SD, (*n* = 3).

| **Blood routine  index** | **Day 0** | **Day 7** | **Day 14** |
| --- | --- | --- | --- |
| WBC(10^9/L) | 10.52±3.07 | 8.7±1.53 | 9.19±1.94 |
| Neu#(10^9/L) | 1.46±0.45 | 1.41±0.41 | 0.93±0.30 |
| Lym#(10^9/L) | 8.36±2.60 | 6.74±0.79 | 7.95±2.37 |
| Mon#(10^9/L) | 0.48±0.07 | 0.34±0.29 | 0.18±0.13 |
| Eos#(10^9/L) | 0.21±0.06 | 0.20±0.06 | 0.12±0.11 |
| Bas#(10^9/L) | 0.01±0.01 | 0.01±0.01 | 0.01±0.00 |
| IMG#(10^9/L) | 0.01±0.01 | 0.02±0.01 | 0.01±0.01 |
| Neu%(10^12/L) | 13.80±1.67 | 16.23±2.15 | 10.73±4.96 |
| Lym% | 79.23±1.45 | 77.97±4.52 | 85.57±7.42 |
| Mon% | 4.77±1.34 | 3.70±2.69 | 2.07±1.86 |
| Eos% | 2.13±0.84 | 2.23±0.25 | 1.53±1.36 |
| Bas% | 0.07±0.06 | 0.07±0.06 | 0.10±0.00 |
| IMG% | 0.10±0.10 | 0.20±0.01 | 0.13±0.15 |
| RBC% | 9.60±0.8 | 9.39±0.18 | 8.78±0.58 |
| HGB% | 144.67±10.07 | 139.67±2.52 | 131.67±8.50 |
| HCT(g/L) | 49.17±3.21 | 47.67±0.42 | 43.60±4.30 |
| MCV(fL) | 51.23±0.50 | 50.80±0.53 | 49.60±2.36 |
| MCH(pg) | 15.10±0.1 | 14.90±0.00 | 15.00±0.36 |
| MCHC(g/L) | 294.33±1.15 | 293.00±3.00 | 302.67±10.60 |
| RDW-CV% | 15.00±0.78 | 15.50±0.10 | 16.17±1.24 |
| RDW-SD(fL) | 26.87±0.59 | 27.23±0.15 | 28.33±1.03 |
| PLT(10^9/L) | 758.33±53.14 | 738.00±35.03 | 780.67±71.25 |
| MPV(fL) | 5.33±0.06 | 5.40±0.00 | 5.60±0.35 |
| PDW | 14.67±0.06 | 14.70±0.10 | 14.80±0.00 |
| PCT% | 0.41±0.03 | 0.40±0.02 | 0.44±0.05 |
| P-LCC(10^9/L) | 30.00±2.65 | 31.33±2.52 | 35.33±4.51 |
| P-LCR% | 3.97±0.65 | 4.23±0.29 | 4.53±0.42 |
|  |  |  |  |

Fig.S6: H&E sections of various organs after intravenously with CTT/MPDA@L-Ser for 7 and 14 days.

Fig.S7 (A) Western Blot imaging and (B) semi-quantitative analysis of Kim-1 protein expression in HK-2 cells induced by DDP. (*n* = 4)

Fig.S8: The viability of HK-2 cells treated with different concentrations of (A) CTT, (B) MPDA, (C) CTT/MPDA NPs and (D) CTT/MPDA@L-Ser NPs.67(*n* = 3).

Fig.S9: The viability of DDP induced HK-2 cells treated with different concentrations of (A) CTT, (B) MPDA, (C) CTT/MPDA NPs and (D) CTT/MPDA@L-Ser NPs. (*n* = 3)

Fig.S10: (A) The viability of DDP induced HK-2 cells treated with Fer-1 and CTT/MPDA@L-Ser NPs. (B) The viability of RSL3 induced HK-2 cells treated with CTT/MPDA@L-Ser NPs. (*n* = 4)

Fig.S11: (A) LSCM images and (B) semi-quantitative fluorescence analysis of DDP induced HK-2 cells after different cell treatments (*n* = 3). (C) Western blot protein expression of GPX4 and (D) semi-quantitative analysis (*n* = 4).

Fig.S12: Western blotting analysis of Kim-1 in mice. Values are expressed as mean ± SD, ******p* < 0.05, *******p* < 0.01, ********p* < 0.001 (*n* = 4).
